# Special Issue: “Antimicrobial Resistance in *Pseudomonas aeruginosa*”

**DOI:** 10.3390/microorganisms11030744

**Published:** 2023-03-14

**Authors:** Sara Hernando-Amado, José Luis Martínez

**Affiliations:** Centro Nacional de Biotecnología, CSIC, Darwin 3, 28043 Madrid, Spain

*Pseudomonas aeruginosa* is one of the most prevalent pathogens causing nosocomial infections, mainly in patients presenting with basal pathologies or those who are immunocompromised [1,2]. In addition, this opportunistic pathogen is a leading cause of chronic infections in patients with cystic fibrosis [3] and chronic obstructive pulmonary disease [4]. The success of *P. aeruginosa* in causing infections, even in patients treated with antibiotics, relies on its intrinsically low susceptibility to antibiotics [5,6,7,8] and its capacity to acquire resistance through both the acquisition of mutations, especially during chronic infections [9], and the acquisition of resistance genes [10]. In addition, its capacity to produce biofilms renders the treatment of infections caused by this bacteria even more difficult [11,12,13]. These are the reasons why *P. aeruginosa* is included in two groups of pathogens, namely, the ESKAPE pathogens and top ten resistant microorganisms, that show high clinical relevance and are considered high-risk concerning antibiotic resistance [14,15,16]. Consequently, the development of new therapeutic strategies against *P. aeruginosa* is urgently needed. For this purpose, the discovery of new antibiotics and inhibitors of bacterial virulence, as well as the improvement of the efficacy of the available antibiotics, is required.

This Special Issue on “Antimicrobial resistance in *Pseudomonas aeruginosa*” presents novel information about intrinsic and acquired resistance in *P. aeruginosa*, as well as novel strategies that may be used to address the antibiotic resistance of this pathogen. Previous work has shown that the PhoP–PhoQ two-component regulatory system is fundamental in establishing resistance to both antimicrobial peptides and aminoglycosides in different organisms, including *P. aeruginosa* [17], by regulating the expression of the *arnBCADTEF-pmrE* operon that encodes enzymes that modify bacterial lipopolysaccharides. The article of Yang B et al. [18] expands on this previous knowledge by identifying novel PhoP–PhoQ-regulated genes that also contribute to the response of *P. aeruginosa* to polymyxin. Among them, *papP* and *slyB* were shown to be involved in the maintenance of outer membrane integrity, while mpl (also involved in ß-lactams’ resistance in *P. aeruginosa* [19]) and *ppgS-ppgH* are involved in the maintenance of inner membrane integrity. In a demonstrative example of whole-genome sequencing for the study of molecular epidemiology, Hernández-García M et al. [20] showed that, while ciprofloxacin resistance in MDR/XDR *P. aeruginosa* clinical isolates is mainly associated with classical mutations in the *gyrA* and *parC* genes, the presence of chromosomal *crpP*-like genes in well-adapted hospital lineages, such as CC175 and CC235, could also contribute to this phenotype. However, in contrast to expectations and despite the fact that *crpP* has been described as a mobile quinolone resistance determinant [21], the presence of CrpP proteins does not always correlate with quinolone resistance. Indeed, among the six analyzed alleles, only one (CrpP6) caused a decrease in susceptibility to quinolones, while the other five presented a missense mutation in an amino acid known to be critical for quinolones’ resistance. The fact that, in most cases, the studied CrpP proteins do not confer resistance to quinolones is in agreement with recent claims stating that CrpP is not a fluoroquinolone-inactivating enzyme, although it can contribute to quinolone resistance when combined with other quinolone resistance mechanisms [22]. Altogether, these findings incite debate about the role of CrpP in clinical resistance to quinolones. However, the possibility that specific alleles (as CrpP6) may emerge and contribute to this phenotype is one that should be explored. The article of Nitz F et al. [23] also explores the molecular epidemiology of *P. aeruginosa* infections, in this case linking the molecular profiles of virulence and antibiotic resistance genes in clinical isolates of *P. aeruginosa*. Interestingly the authors showed that three of the ninety-nine analyzed isolates presented the oxacillin resistance genes *bla_OXA-23_* and *bla-_OXA51_*, this being the first report of the presence of these enzymes in *P. aeruginosa*. The prevalence of several of the studied virulence genes among the different isolates was very high, a feature that is in agreement with previous studies on the population genetics of *P. aeruginosa* [24,25], indicating that several *P. aeruginosa* virulence determinants belong to its core genome. However, virulence determinants such as *apr* or *nan-1* are present as just a small fraction of isolates, suggesting a more recent acquisition. An aspect that remains to be established is the extent to which these “low prevalent” virulence determinants modulate the virulence capacity of *P. aeruginosa*. Finally, this Special Issue also aimed to identify novel therapies against this pathogen, especially against biofilms produced by *P. aeruginosa*, which cause remarkable tolerance to antibiotics. Li Y and Wu MX [26] identified a combination therapy comprising 405 nm blue light, carvacrol (a phenolic monoterpenoid present in essential oils from aromatic plants), and ciprofloxacin which synergistically killed polymicrobial biofilms containing, in addition to *P. aeruginosa*, other species such as *Staphylococcus aureus* and *Acinetobacter baumannii*. The use of blue light as a therapy for treating infections is an area that has been under development in recent years. The benefit of blue light compared to other light-based microbicides is that it does not require exogenous photosensitizers. It excites bacterial chromophores independently, generating microbicidal reactive oxygen species [27]. Previous studies showed that carvacol strongly increases blue-light-mediated oxidative burst [28]. The work of Li Y and Wu MX [26] shows that the combination of blue light and carvacol increases membrane permeability, facilitating the activity of ciprofloxacin against refractory biofilms. The authors propose that these findings could serve in the design of antimicrobial dressings for wound infections that contain quinolones and carvacol and are based on light-transmissible materials. If feasible, this could mark a relevant therapeutic advance, taking into consideration the fact that ciprofloxacin is not effective against chronic wound biofilms [29].

Tackling antibiotic resistance requires detailed epidemiological knowledge of the population dynamics and the antibiotic resistance determinants of bacterial pathogens, as well as new approaches that can be used to fight resistance. These approaches can be based on the development of novel antimicrobials or a better use of those already available. Combination therapy, based on the exploitation of evolutionary trade-offs [30,31], or the use of adjuvants that occasionally show antimicrobial activity, as described by Li Y and Wu MX [26], will certainly help in this endeavor.

Finally, although this Special Issue does not focus on works that use evolutionary knowledge to design approaches which make a better use of the available antibiotics, it is important to highlight the enormous importance that this type of approach will have in efforts to manage bacterial infections more rationally and efficiently. For this purpose, the identification of robust evolutionary trade-offs (such as collateral sensitivity) will be critical.
